# New Brush-Type Chiral Stationary Phases for Enantioseparation of Pharmaceutical Drugs

**DOI:** 10.3390/molecules24040823

**Published:** 2019-02-25

**Authors:** Anamarija Knežević, Jurica Novak, Vladimir Vinković

**Affiliations:** 1 Division of Organic Chemistry and Biochemistry, Ruđer Bošković Institute, Bijenička cesta 54, Zagreb 10000, Croatia; vvink@irb.hr; 2 Division of Physical Chemistry, Ruđer Bošković Institute, Bijenička cesta 54, Zagreb 10000, Croatia; Jurica.Novak@irb.hr; 3 South Ural State University, 20-A, Tchaikovsky Str., Chelyabinsk 454080, Russia

**Keywords:** chiral chromatography, chiral recognition, intermolecular interactions, chiral drugs, Whelk-O1 column, mobile phase additives

## Abstract

The importance of chirality in drug development is unquestionable, with chiral liquid chromatography (LC) being the most adequate technique for its analysis. Among the various types of chiral stationary phases (CSPs) for LC, brush-type CSPs provide the base for interaction analysis of CSPs and enantiomers, which provide valuable results that can be applied to interaction studies of other CSP types. In order to analyze the influence of aromatic interactions in chiral recognition, we designed a set of ten new brush-type CSPs based on (*S*)-*N*-(1-aryl-propyl)-3,5-dinitrobenzamides which differ in the aromatic unit directly linked to the chiral center. Thirty diverse racemates, including several nonsteroidal anti-inflammatory drugs and 3-hydroxybenzodiazepine drugs, were used to evaluate the prepared CSPs. Chromatographic analysis showed that the three new CSPs separate enantiomers of a wide range of compounds and their chromatographic behavior is comparable to the most versatile brush-type CSP—Whelk-O1. The critical role of the nonbonding interactions in positioning of the analyte (naproxen) in the cleft of **CSP-6**, as well as the analysis of interactions that make enantioseparation possible, were elucidated using computational methods. Furthermore, the influence of acetic acid as a mobile phase additive, on this enantiorecognition process was corroborated by calculations.

## 1. Introduction

Chirality is an essential property in the development of pharmaceutical drugs, as well as in agrochemistry, food science, etc. It was put into the foreground in 1992 when the FDA issued its policy statement concerning the development of stereoisomeric drugs [1]. Even though the development of enantiopure compounds was not mandatory, it became the de facto standard in the pharmaceutical industry, also enabling the new possibilities in drug development, i.e., the chiral switch concept [2]. Accordingly, the need for fast, simple and reliable methods for separation of enantiomers, determination of enantiomeric excess (*ee*) and absolute configuration (AC) has increased. Although chiral liquid chromatography (chiral LC) has a great influence on the determination of absolute configuration [3], its main role is being the most powerful method for the separation of enantiomers [4] and for measuring the enantiopurity of organic compounds. Since the 1990s, when the availability of chiral stationary phases (CSPs) became widespread, chiral LC using CSPs has been the most widely used technique for chiral separations [5]. Besides being simple, versatile and reliable method, chiral LC has also became an ultrafast technique with enantioseparations in the sub-minute time frame [6,7,8,9,10,11,12].

Over time, various types of CSPs have been developed, including more than a hundred which have been commercialized. Their properties and mechanism of enantioseparation depend on the nature of the chiral selector [5,13]. Polysaccharide-based CSPs are the most broadly applied for chiral LC separations [14]. However, there are also several Pirkle-type (or brush-type) CSPs, e.g., Whelk-O1 and ULMO, whose versatility has enabled their wide application [13,15]. These CSPs, which consist of small organic molecules covalently bound to the support, are also the most widely investigated CSPs regarding the chiral recognition mechanism [13,16]. The understanding of chiral separation mechanism is essential for estimating elution order, predicting the types of analytes which can be separated on a certain CSP, and improving the design of new selectors. Clearly, the enantioseparation process is easier to elucidate by analyzing interactions of the analyte and a small selector compared to polysaccharide CSPs, whether using experimental or computational methods [5]. Chiral recognition of brush-type CSPs is based on well-documented interactions between the selector and enantiomers of the analyte—hydrogen bonds, dipole-dipole interactions, van der Waals interactions and, in particular, aromatic interactions [16].

**Figure 1:**
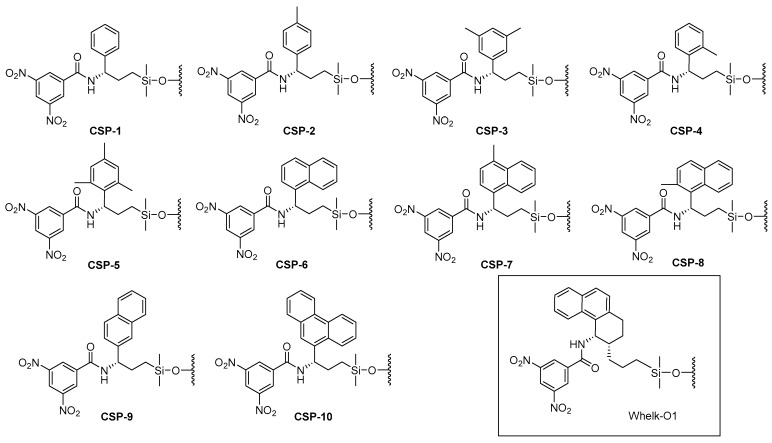
Structures of new brush-type CSPs and Whelk-O1.

The goal of this research was threefold. First, ten new brush-type CSPs were prepared, with a molecular design based on previous studies of chiral recognition in CSP [13,17,18,19]. The objective was to obtain a CSP with a versatility as similar as possible to the Whelk-O1 column, the brush-type CSP with the broadest application in industry and academia. We opted for a structure with one amide bond, analogous to the Whelk-O1 column, and two aromatic groups—a 3,5-DNB aromatic unit and a substituted aromatic moiety (Figure 1). We tested the prepared CSPs using thirty diverse racemates and determined the CSPs with the best performance. Second, the variability of the aromatic moiety enabled us to elucidate the influence of aromatic substituent on the enantiorecognition process. Keeping that in mind, we investigated the role of nonbonding interactions relevant for chiral recognition on CSPs using computational methods, with special attention to hydrogen bond networks and aromatic interactions. Third, we explored the influence of acetic acid as an additive in the mobile phase using extensive ab initio methods. To the best of our knowledge, this is the first study that examines the possibility of positioning a minor additive within the chiral binding site and its influence on enantiospecific interactions.

## 2. Results and Discussion

### 2.1. Preparation of New Brush-Type Chiral Stationary Phases (CSPs)

We previously described the synthesis and determination of an absolute configuration of (*S*)-*N*-(1-aryl-allyl)-3,5-dinitrobenzamides (**DNB-1**–**DNB-10**) [20]. These compounds are excellent candidates for the preparation of a new Pirkle-type CSPs for HPLC since they possess all properties of a good selector—they are rigid and contain a strong π-acceptor acid (DNB) as well as a π-donor base aromatic group. Furthermore, allyl group in the structure of DNBs enables their binding to silica gel using a simple and straightforward procedure.

**Scheme 1:**
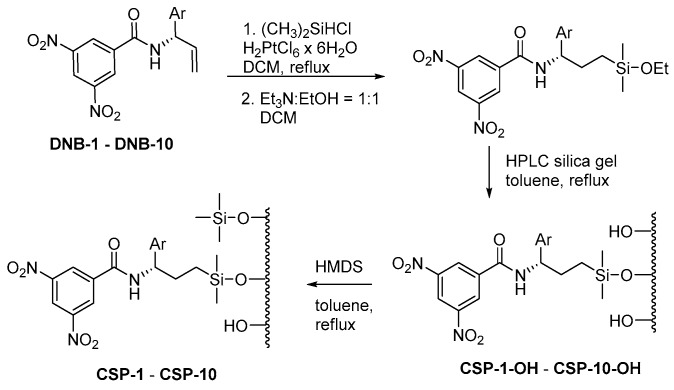
Preparation of new brush-type chiral stationary phases (CSPs) from corresponding (*S*)-*N*-(1-aryl-allyl)-3,5-dinitrobenzamides (DNBs).

For the binding of the chiral DNB selectors onto silica gel we chose a method that does not introduce additional polar moieties into the CSP structure (Scheme 1) [21]. Hydrosilylation of DNBs was conducted using chlorodimethylsilane and Speier’s catalyst, followed by the replacement of a chloride with an ethoxy group using trimethylamine and ethanol. Obtained compounds were passed through short silica column and attached to 5 μm silica gel in boiling toluene. Before the end-capping procedure, we analyzed prepared CSPs using elemental analysis and infrared spectroscopy in order to determine the amount of selector attached onto the silica gel. We performed end-capping in the last step to protect free silanol groups with hexamethyldisilazane [22]. Ten new CSPs were thus prepared from the corresponding DNBs (Figure 1) with the optimal 0.2 mmol selector per 1 g of CSP loading [21]. The as-prepared CSPs were finally packed in steel columns using the slurry packing technique.

### 2.2. Evaluation of Prepared CSPs

**Figure 2:**
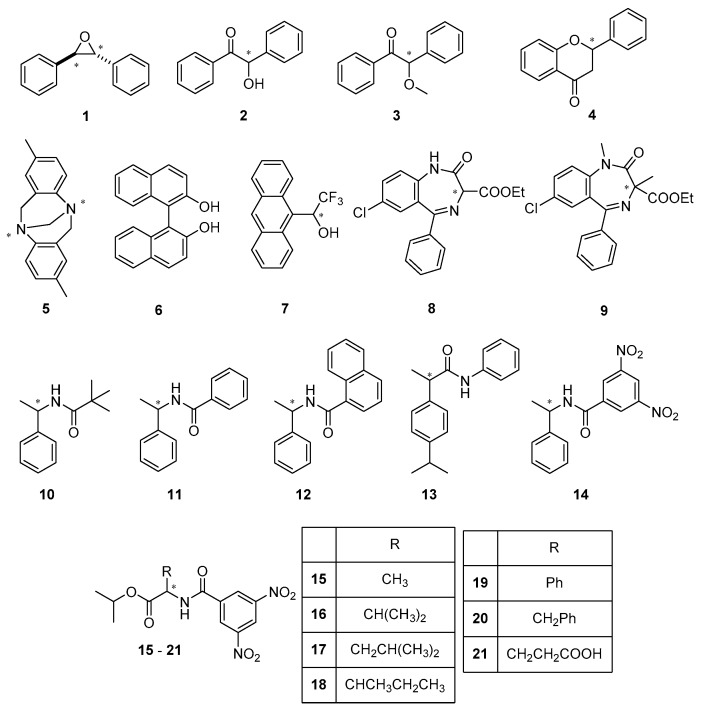
Structures of the racemic analytes used for the evaluation of prepared CSPs.

In order to test the performance of the prepared CSPs we used a diverse set of analytes (Figure 2) which is usually used by our group to evaluate new CSPs [18,19]. The evaluation of prepared CSPs was performed in normal phase chromatography mode using the mixtures of hexane and 2-propanol as the mobile phase at room temperature and with UV detection at 254 nm. Compounds **1**–**7** and **9** were analyzed in the mobile phase containing 10% of 2-propanol in hexane, while the mobile phase for the analysis of compounds **8** and **10**–**21** contained 20% of 2-propanol due to their longer retention times. Our goal was to evaluate CSPs with respect to their difference in molecular structure and consequently to neglect the influence of quality of the column packing, column dimensions, etc. Therefore the relevant value for consideration is the separation factor (selectivity, α), which depends the most on the structure of the CSP and has the greatest influence on overall resolution.

Tested set of compounds can be subdivided into four subsets. Compounds **1**–**4** are small molecules with one or none carbonyl groups which could be the acceptor but not the donor of hydrogen bonds. Because of these characteristics, the satisfactory enantioseparation of compounds **1**–**4** is usually hard to achieve on brush-type CSPs. The second subset consists of compounds **5**–**9** which possess bigger aromatic groups and hydroxyl or amino groups capable of hydrogen bond formation. These compounds are usually well separated on brush-type CSPs. Finally, there are two subsets consisting of aromatic amides, **10**–**21**, with the last subset (**15**–**21**) also bearing an ester group. These compounds can form strong hydrogen bonds and aromatic interactions which makes them easier to separate using brush-type CSPs.

**Figure 3:**
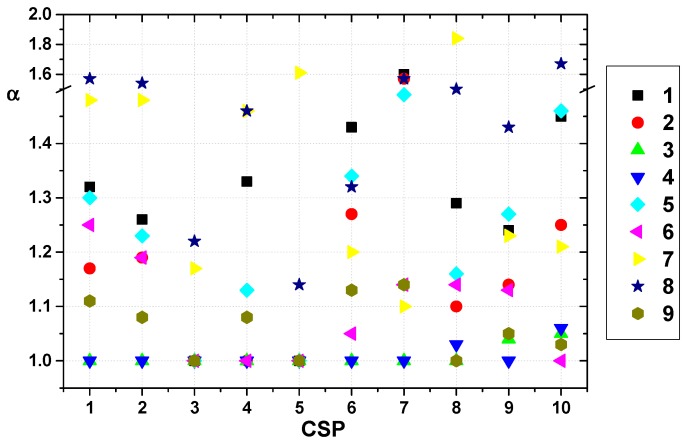
Separation factors of compounds **1**–**9** on prepared **CSP-1**–**CSP-10**, mobile phase hexane-2-propanol = 90:10. For compound **8**, the mobile phase ratio was 80:20.

The results indicate that CSPs which contain substituted phenyl aromatic groups, in most cases do not resolve compounds **1**–**4** (Figure 3). Enantioseparation of these compounds is better using the naphthyl series of CSPs, although the separation of compounds **3** and **4** is achieved on just a few CSPs. In general, enantioseparation results obtained for compounds **5**–**9** are better. Overall, the lowest separation factor is observed with CSPs like **CSP-3**, **CSP-4** and **CSP-5**, which have more than one methyl substituent or methyl at the *ortho* position of the phenyl core. 

**Figure 4:**
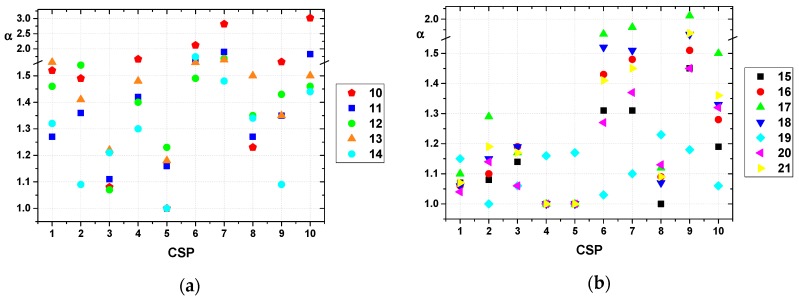
Separation factors of compounds **10**–**14** (**a**) and **15**–**21** (**b**) on prepared **CSP-1**–**CSP-10**, mobile phase hexane-2-propanol = 80:20.

Compounds **10**–**14** are resolved well using all CSPs, with the only exceptions being **CSP-3** and **CSP-5** (Figure 4a). These CSPs demonstrate the lowest separation factors due to their phenyl aromatic group in the combination with two or three methyl substituents near the chiral carbon atom, which disturb the enantiorecognition process due to steric hindrance. Also, the retention times of these compounds are shorter for the phenyl series of CSPs than the naphthyl series (Table S2). This means that interactions are weaker, as expected for smaller aromatic systems.

The initial assumption was that 3,5-dinitrobenzoyl derivatives of amino acids with an isopropyl protecting group **15**–**21** would be resolved well on the prepared CSPs since they possess aromatic, ester and amide groups potentially capable of forming interactions with CSPs which may result in chiral recognition. However, the difference between the phenyl series of CSPs and larger aromatic substituents is even more pronounced in these examples (Figure 4b). Here, it is demonstrated that only CSPs with larger aromatic groups (naphthyl and phenantryl) show very good enantio-separations of these compounds. The lowest results in the naphthyl series were obtained for **CSP-8** which possesses a methyl group at position 2 of the naphthyl ring.

**Figure 5:**
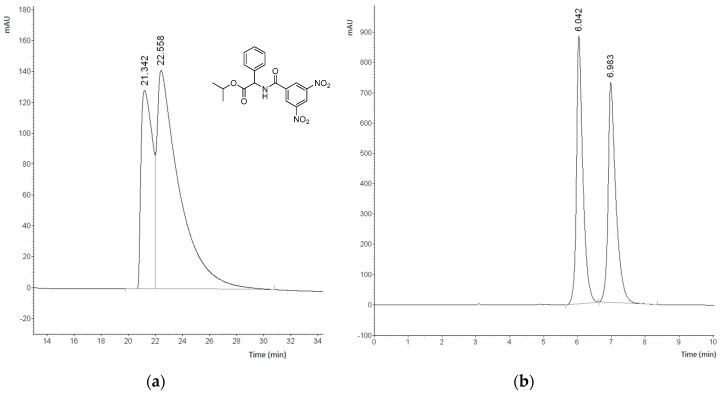
Chromatogram of the separation of racemate **19** on **CSP-10** (**a**) and **CSP-8** (**b**); mobile phase hexane-2-propanol = 80:20, flow 1mL/min, UV detection at 254 nm.

It is interesting to point out that the enantioseparation of racemate **19** on the prepared CSPs is quite unexpected (Figure 5). On the CSPs which show the overall lowest enantioseparation capabilities, its separation factor is the highest in the **15**–**21** subset. On the contrary, on the CSPs that demonstrate the best performance, this separation factor is the lowest of the subset. This result exemplifies how chiral recognition is difficult to predict and often depends on subtle details.

**Figure 6:**
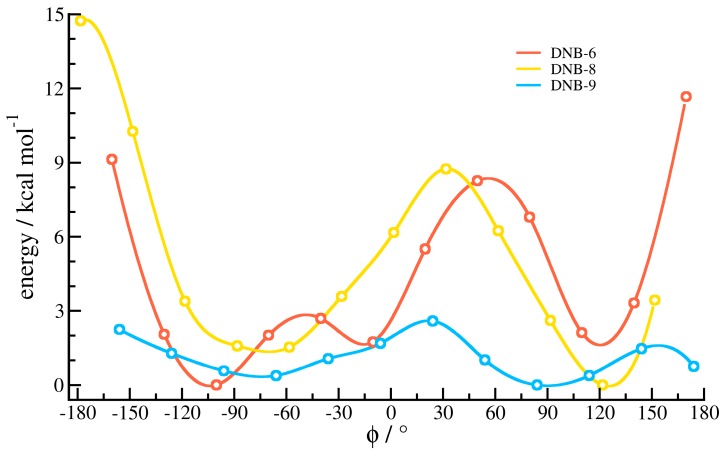
Relaxed potential energy surface scan around C*-C^α^ bond of **DNB-6** (red), **DNB-8** (yellow) and **DNB-9** (blue). Dihedral angle Φ is defined by atoms C^β^-C^α^-C*-N. Calculations were performed on M06-2X/aug-cc-pVDZ level of theory.

The above presented results demonstrate that both size and substitution of aromatic group play an important role in the enantioseparation capabilities of prepared CSP. Larger aromatic groups (naphthyl and phenanthryl) overall display higher separation factors for the tested set of racemates. This is expected since intermolecular aromatic interactions, which positively influence the chiral recognition, are stronger for larger aromatic groups. Substitution of more than one methyl group on the phenyl ring decreases the separation factor for all the tested racemates. On the other hand, the influence of the monosubstitution of the aromatic group shows two opposite trends. When the substitution is at the 4-position, there is no noticeable difference compared to the aromatic group without substituents (**CSP-1** compared to **CSP-2** and **CSP-6** to **CSP-7**). Contrarily, substitution at the 2-position greatly influences the enantioseparation capabilities of the prepared CSPs (**CSP-2** compared to **CSP-4** and **CSP-7** to **CSP-8**). The methyl substituent in this case is near the chiral center and can influence the chiral recognition in two ways—it sterically hinders the chiral center which decreases enantiorecognition and it increases the rigidity of the CSPs by reducing the rotational freedom around C*-C^Ar^ bond (Figure 6). The influence of the rigidity of CSP on enantioseparation capability is substantial. **CSP-9** compared to **CSP-6** demonstrates higher flexibility and somewhat lower enantioseparation characteristics (see Figure 3 and Figure 4). On the other hand, **CSP-8** compared to **CSP-6** has higher rigidity and considerably decreased enantioseparation results (which are partially due to steric hindrance of the chiral center). This indicates that a good CSP should be sufficiently rigid to strongly interact with one sterically compatible enantiomer, but also flexible enough to accommodate the analyte and achieve a maximum number of interactions for a wide range of compounds.

### 2.3. Enantioseparation of Pharmaceutical Drugs on Prepared CSPs

**Figure 7:**
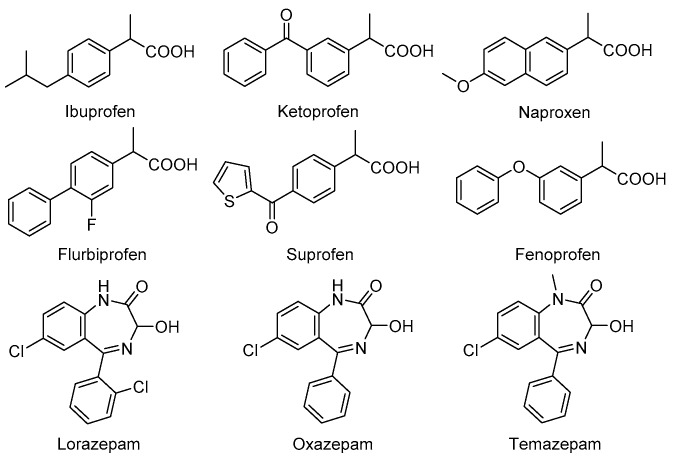
Structures of the tested non-steroidal anti-inflammatory drugs (NSAIDs) and 3-hydroxy-benzodiazepine drugs.

Since CSPs bearing naphthyl and phenanthryl aromatic groups were demonstrated as more versatile than phenyl CSPs, **CSP-6**–**CSP-10** were chosen for testing the enantioseparation of several pharmaceutical drugs including non-steroidal anti-inflammatory drugs (NSAIDs) and 3-hydroxy-benzodiazepine drugs (Figure 7).

**Figure 8:**
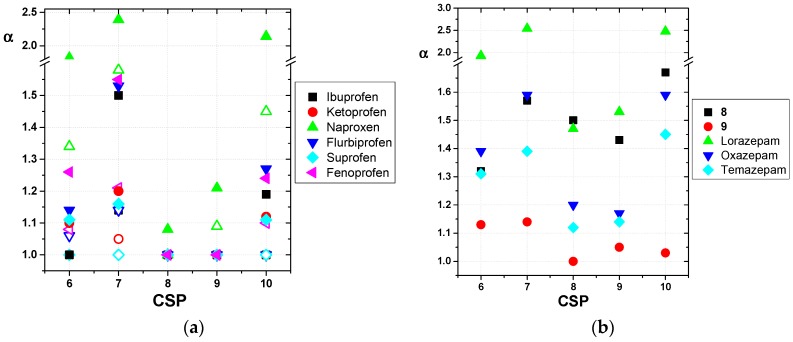
(**a**) Separation factor of NSAIDs on prepared **CSP-6**–**CSP-10** using a mobile phase hexane-2-propanol-CH_3_COOH = 90:10:0.1 (hollow symbols) and hexane-2-propanol = 80:20 + 1 g dm^−3^ NH_4_OAc (filled symbols); (**b**) Separation factor of compounds **8**, **9** and 3-hydroxy-benzodiazepine drugs on prepared **CSP-6**–**CSP-10** using a mobile phase hexane-2-propanol = 80:20 + 1 g dm^−3^ NH_4_OAc.

Given that NSAIDs are organic acids, acetic acid (0.1%) was selected as the mobile phase additive. Acidic additives are frequently used in the mobile phases for the analysis of acidic analytes because of their positive influence on the peak shape and retention time [23]. Results of the enantioseparation of NSAIDs on the prepared **CSP-6**–**CSP-10** (Figure 8a) showed that only naproxen is resolved well on prepared CSPs, while some of the drugs from our test set are only partially resolved on **CSP-7**.

**Figure 9:**
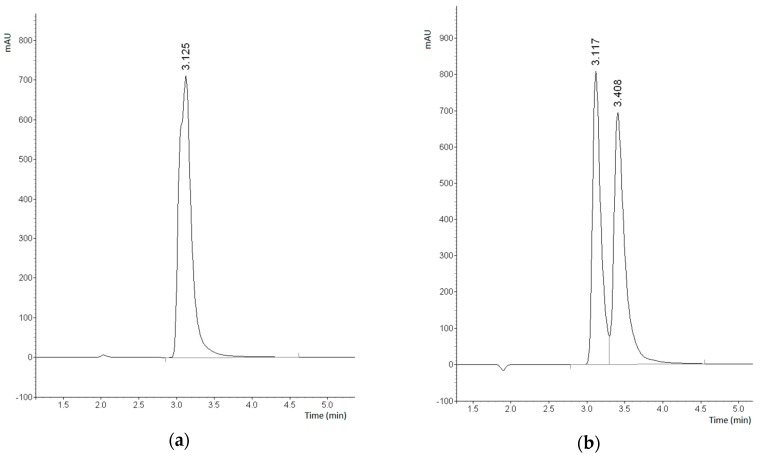
Chromatograms of the separation of flurbiprofen on **CSP-10** using mobile phase: hexane-2-propanol-CH_3_COOH = 90:10:0.1 (**a**) and hexane-2-propanol = 80:20 + 1 g dm^−3^ NH_4_OAc (**b**).

It was demonstrated that in some cases neutral salts, such as ammonium acetate or ammonium formate, have a positive influence on the enantioseparation of racemates [23]. For example, NSAIDs showed excellent enantioseparation results on Whelk-O1 columns using a mobile phase with ammonium acetate as an additive [24]. Therefore, we investigated the enantioseparation performance of the newly prepared **CSP-6**–**CSP-10** on NSAIDs also using ammonium acetate as a mobile phase additive (Figure 8a). Although **CSP-8** and **CSP-9** still only separate the enantiomers of naproxen, the performance of the remaining CSPs was substantially improved. This is especially evident in the case of **CSP-10** (Figure 9) which can separate all of the tested NSAIDs using the abovementioned conditions.

Compounds **8** and **9**, which are structural analogs of 3-hydroxybenzodiazepines with an ester group at the 3-position, were resolved well using the prepared CSPs. Therefore, we decided to test the naphthyl series of CSPs for the enantioseparation of 3-hydroxybenzodiazepine drugs (Figure 8b). Since these compounds possess acidic and basic groups, ammonium acetate was also used as a mobile phase additive. 3-Hydroxybenzodiazepine drugs show very good enantioseparation results on the prepared CSPs, with the best results obtained on **CSP-6**, **CSP-7** and **CSP-10**.

Overall, the best separation factors for the enantioseparation of the tested drugs were obtained on **CSP-6**, **CSP-7** and **CSP-10**. Given the importance of these compounds in the pharmaceutical industry, their enantioseparation was previously analyzed on several commercial brush-type CSP, including Whelk-O1 [24]. In order to compare the results obtained on our CSP with the results obtained for Whelk-O1, we analyzed these pharmaceutical drugs using the same hexane—2-propanol = 80:20 + 1 g dm^−3^ NH_4_OAc mobile phase (Table 1).

From the obtained results it is evident that these three new CSPs have promising enatioseparation capabilities which are comparable to those of Whelk-O1. Furthermore, it must be emphasized that the column Whelk-O1, being a commercial one, is technologically optimized regarding the choice of silica gel used as the support, the binding procedure, as well as the packing procedure. It is known that all of these conditions influence the enantioseparation capabilities of CSPs [25,26]. The prepared CSPs haven’t been optimized at this point, leaving plenty of room for improvement of these CSPs. These columns have one more advantage over Whelk-O1. In order to prepare Whelk-O1 enantiopure selector, preparative chiral chromatography must be used which has low productivity due to the low solubility of the compound [27,28]. On the other hand, the enantiopure selectors of our CSPs (**DNB-6**, **DNB-7** and **DNB-10**) can be obtained by enzyme resolution using a cheap commercial lipase—*Candida antarctica* lipase B (CAL-B) [20,29].

### 2.4. Analysis of Interactions Responsible for Chiral Recognition between Naproxen and ***CSP-6***

To explore the nature of chiral binding site, a set of molecular dynamics simulations in vacuum of **CSP-6** with naproxen was performed. Since we are interested in distinguishing the details that enable enantiomeric separation, simulations were run separately for (*S*)- and (*R*)-enantiomers of naproxen. According to a ^1^H-NMR study of chiral recognition of (*S*)- and (*R*)-naproxen in the presence of Whelk-O1 selector analog [30] and to the molecular dynamics simulations of the interface of modified Whelk-O1 selector and naproxen in *n*-hexane [31], both enantiomers of naproxen dock inside the cleft, most probably by an M1 mechanism [32,33]. The main feature of the M1 mechanism is a hydrogen bond between the drug and the amide hydrogen, while aromatic interactions with the dinitrophenyl moiety introduce additional stability to the complex. For simplicity in our computational models we have neglected the non-polar solvent and a linker, the interface to silica gel, substituting it with a hydrogen atom.

**Figure 10:**
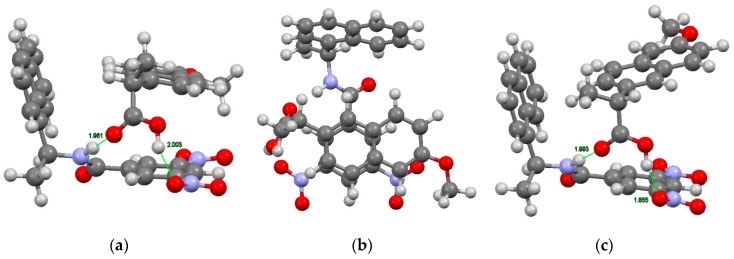
The lowest energy structures of (*S*)-**A** (**a**), (*S*)-**A**, top view (**b**) and (*R*)-**A** (**c**). Selected bond lengths are indicated in Å. B3LYP/def2-TZVPP//M06-2X/def2-TZVPP (COSMO, ε = 2.78).

The complex of **CSP-6** and (*S*)-naproxen ((*S*)-**A**) has lower energy (by 1.8 kcal mol^−1^) than the lowest energy complex of **CSP-6** and (*R*)-naproxen ((*R*)-**A**) (Figure 10). This result is in accordance with the experimental results which demonstrate that (*R*)-naproxen is the first eluting enantiomer on **CSP-6** (Figure S1). Ten additional complexes within 2.9 kcal mol^−1^ were examined (Figure S2). In (*S*)-A complex the naphthyl and DNB subunits are almost perpendicular to each other. Naproxen is hydrogen bonded to the amide hydrogen (2.00 Å), with the carbonyl oxygen being a H-bond acceptor and in the vicinity of the β-hydrogen of DNB, being only 2.44 Å apart. The second most important H-bridge is between an oxygen of the DNB nitro group and the hydrogen from the naproxen carboxyl group. The weakest interaction of this type is responsible for anchoring the opposite side of naproxen, the methoxy moiety, to the second nitro group of the DNB subunit. The parallel face-centered stacking arrangement between the two aromatic rings with the distance between the centroids of the naproxen and DNB rings of 4.11 Å, represents a good example of aromatic interaction between strongly electron-deficient (DNB) and neutral aromatic rings (Figure 10b). The next intriguing space oriented interaction is the H-aromatic interaction [34], where three hydrogens of naproxen, including the hydrogen on the chiral carbon atom, are oriented toward the naphthyl ring, forming a recognizable T-shaped motif whose rings’ planes form an angle of 75°. Alternative modes of complexation are also found, like the one where naproxen is outside the cleft ((*S*)-**A**-4) or a complex where naproxen is hydrogen bonded to the carbonyl oxygen of **CSP-6** ((*S*)-**A**-5). Those complexes are 2.5 kcal mol^−1^ higher in energy than (*S*)-**A**, and will not be discussed further.

Just by reversing groups at the chiral carbon on naproxen in the (*S*)-**A** complex and running optimization, a new complex is identified, (*R*)-**A**-2, 2.8 kcal mol^−1^ higher in energy (see Supplementary Materials). The O-H···O bond is shorter by 0.10 Å, while O···H-N is 0.03 Å longer than the analogous bonds in (*S*)-**A**, but the orientation of the methyl group toward the naphthyl part of **CSP-6** perturbs the parallel face-centered stacking arrangement almost perfectly possible in the (*S*)-enantiomeric complex. The H-aromatic interaction network is also perturbed, which can be seen from the fact that only hydrogens from the α ring are pointing toward naphthyl moiety.

The (*R*)-**A** complex, more stable than (*R*)-**A**-2 by 0.9 kcal mol^−1^, is characterized by even stronger hydrogen bonds between the naproxen carboxyl group and DNB (1.86 Å) and the amide hydrogen (1.99 Å). The anchoring interaction between the methoxy hydrogen and the nitro group is missing, which is reflected in the increased distance between the centroids of an aromatic ring of naproxen and the DNB subunit to 5.29 Å. By comparing the (*R*)-**A** and (*R*)-**A**-2 structures it is possible to conclude that the main contribution to the stabilization of the complexes comes from hydrogen bonding with aromatic and H-aromatic interactions playing a minor, but significant role in the enantiorecognition process.

Our findings are in agreement with similar theoretical studies on the chiral recognition of Whelk-O1. For a series of complexes of naproxen with Whelk-O1 and modified CSPs, the M1 mechanism is identified as the most probable docking mechanism [31]. Furthermore, the formation of the hydrogen bond was found to be critical for the successful separation of enantiomers [35].

### 2.5. Influence of Acetic Acid as the Mobile Phase Additive on The Enantioseparation of Naproxen on ***CSP-6***

Although it is empirically known that minor additives (organic acids, bases or neutral salts) may have positive effect on the shape of peaks, retention times and selectivity [23], experimental studies on the mechanism of how additives influence enantioseparation are scarce. Several systematic experimental studies were performed, mostly on polysaccharide CSPs under normal phase conditions [36,37,38], as well as using polar organic mobile phases [38,39,40]. Generally, the consensus is that additives in the mobile phase improve the solubility of acidic analytes in nonpolar solvents and suppress non-chiral interactions of analyte with the CSP surface (masking effect), and therefore reduce tailing and can increase selectivity. However, an explanation for the way in which additives could affect the chiral recognition process between the analyte and CSP remains elusive. Some studies [36,39] hypothesize that additives have an ability to displace the analyte or to influence the selector-analyte complex by interfering with the interactions that stabilize the complex, however, these speculative explanations of experimental results have not been substantiated so far.

Our goal was to investigate the possibility of influence of additives on intermolecular interactions between analyte and CSP during enantiorecognition. We opted for acetic acid, a simple acidic additive, and a system we had already investigated—naproxen and **CSP-6**. Acetic acid is known to form dimers in the gas phase and in nonpolar solvents characterized by a strong double hydrogen bond [41,42]. Since naproxen primarily interacts via its carboxylic group with CSPs, we explored the possibility of an interplay of naproxen, acetic acid and **CSP-6**.

**Figure 11:**
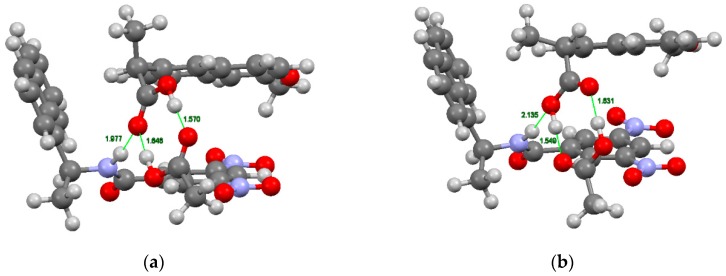
The lowest energy structures of (*S*)-**B** (**a**) and (*R*)-**B** (**b**). Selected bond lengths are indicated in Å. B3LYP/def2-TZVPP//M06-2X/def2-TZVPP (COSMO, ε = 2.78).

The most stabile complex of naproxen, acetic acid and **CSP-6** is (*S*)-**B**, which is 3.9 kcal mol^−1^ lower in energy than (*R*)-**B** (Figure 11). A common motif of both structures is an 8-membered ring formed by a double hydrogen bridge connecting acetic acid and the carboxylic acid group of naproxen. In the (*S*)-**B** complex, the N-H···O bond length is 2.90 Å, while naproxen’s carbonyl oxygen serves as a double H-bond acceptor, associating with acetic acid as well. The analogous bridge to (*R*)-naproxen in (*R*)-**B** is longer (3.14 Å) and of a different nature – the H-acceptor is a carboxylic acid oxygen, which is simultaneously an H-donor to acetic acid. Although acetic acid is not connected to naproxen via enantioselective interactions, it modifies H-bond network compared to a complex without the acid ((*S*)-**A** and (*R*)-**A**). Firstly, the interaction between the carboxylate hydrogen and the nitro group is missing. Secondly, the methyl group of acetic acid is a weak H-donor to the nitro subunit. For (*R*)-**B**, the presence of additive makes the contact between naproxen and **CSP-6** along the carboxyl oxygen more favorable.

Competitive interaction patterns are also found (Figure S3, Supplementary Materials). Only one structure is missing an 8-membered double H-bond ring ((*R*)-**B**-2) where a more complex H-bond network can be seen. An amide is connected to acetic acid, which is a H-donor to naproxen, whose carboxylic acid group is an H-donor to the carbonyl oxygen of **CSP-6**. Again, naproxen is positioned above the DNB subunit but outside the cleft. 

Computational insights into the influence of acetic acid on the enantioseparation of NSAIDs, like naproxen, reveal that additives can interact with both CSP and carboxylic acid analytes. Even non-enantiospecific interactions with the carboxylate moiety of the drug change the interaction arrangements. In the system we studied, the entire binding scheme is shuffled, which is reflected in the increase of N-H···O distance and weakening of both the hydrogen bond and aromatic interactions. The acetic acid also influences the energetics of complexes, increasing the energy difference between the most stable (*S*)- and (*R*)-complex, enabling better separation. Our theoretical consideration of complexes of naproxen and CSPs, with and without acetic acid, indicate that higher selectivity is possible due to interactions of acetic additive with the analyte and CSP.

## 3. Materials and Methods

### 3.1. General Information

All the solvents were puriss. p.a. or HPLC grade and were used directly as supplied by Sigma-Aldrich Chemie GmbH (Munich, Germany), Alfa Aesar (Karlsruhe, Germany) or Acros (part of Thermo Fisher Scientific, Geel, Belgium). For the preparation of chiral stationary phases HPLC silica gel Separon SGX particle size 5 μm and pore size 80 Å from Tessek Ltd. (Prague, Czech Republic) was used. IR spectra were recorded on an MB102 instrument (ABB Bomem, Zurich, Switzerland). Elemental analyses were done on a 2400 CHNS Elemental Analyzer (Perkin-Elmer, Waltham, MA, USA). Chiral HPLC analysis were performed using a Prominence System (Pump LC-20AT, DGU-20A5 Degasser, UV detector SPD-20A, Shimadzu, Kyoto, Japan) or a Knauer system (Pump Knauer 64, 4-Port Knauer Degasser, UV detector Knauer Variable Wavelength Monitor, Interface Knauer, Knauer, Berlin, Germany, and a CD-2095 detector, Jasco, Easton, MD, USA). The packing of prepared CSP into stainless steel columns (150 × 4.6 mm I.D.) was achieved using Knauer Pneumatic HPLC Pump. The (*S*,*S*)-Whelk-O1 column, Regis Technologies, Inc. (Morton Grove, IL, USA), was 5 µm particle size and had dimensions of 250 × 4.6 mm I.D. 

Racemates **8**–**21** used for the evaluation of prepared columns were previously synthesized in the Laboratory for Stereoselective Catalysis and Biocatalysis at the Ruđer Bošković Institute [18,19]. All other compounds were purchased from commercial sources and used without further purification. Unless otherwise noted, the analyses of abovementioned racemates were performed at room temperature using the flow of 1 mL/min and UV detection of the compounds at 254 nm.

### 3.2. General Procedure for the Preparation of CSP-OHs

The corresponding DNB amide (0.75 mmol) was suspended in dry DCM (10 mL) under an inert atmosphere. A solution of hexachloroplatinic(IV) acid hydrate (20 mg) in isopropanol (0.5 mL) was added to the reaction mixture, followed by chlorodimethylsilane (10 mL). The mixture was refluxed for 5 h, cooled to room temperature and concentrated under reduced pressure. Dark residue was once again dissolved in DCM (5 mL), concentrated under reduced pressure and used without further purification. To the solution of the crude product in dry DCM (8 mL) under inert atmosphere, the 1:1 mixture of triethylamine and absolute ethanol (10 mL) was added dropwise and stirred at room temperature for half an hour. The solvent was evaporated to give a dark residue which was filtered through a short column of silica gel (eluent DCM-MeOH = 100:1). The resulting yellow residue was dissolved in dry toluene (5 mL) and added to a suspension of 5 μm HPLC silica gel (1.50 g) in dry toluene (50 mL). The silica gel was dried prior use for 24 h in Dean-Stark apparatus. The suspension was refluxed for 24 h, then filtered using a G4 sinter and washed with toluene (30 mL) and methanol (2 × 30 mL). Prepared CSP-OHs were dried for 4 h at 60 °C and elemental analysis results and IR spectra were recorded.

**CSP-1-OH** CHN analysis: C 5.05; H 0.63; N 1.11 (0.23 mmol/1 g); IR (ν/cm^−1^): 3442 (SiO_2_), 1642, 1540, 1343, 1220–1031 (SiO_2_), 807 (SiO_2_), 465 (SiO_2_).

**CSP-2-OH** CHN analysis: C 4.20; H 0.85; N 0.62 (0.18 mmol/1 g); IR (ν/cm^−1^): 3450 (SiO_2_), 1641, 1544, 1346, 1219–1034 (SiO_2_), 807 (SiO_2_), 731, 466 (SiO_2_).

**CSP-3-OH** CHN analysis: C 4.91; H 0.93; N 0.75 (0.20 mmol/1 g); IR (ν/cm^−1^): 3434 (SiO_2_), 1640, 1539, 1219–1034 (SiO_2_), 808 (SiO_2_), 464 (SiO_2_).

**CSP-4-OH** CHN analysis: C 5.39; H 0.82; N 0.51 (0.24 mmol/1 g); IR (ν/cm^−1^): 3439 (SiO_2_), 1640, 1542, 1349, 1227–1030 (SiO_2_), 808 (SiO_2_), 731, 465 (SiO_2_).

**CSP-5-OH** CHN analysis: C 5.43; H 0.80; N 0.70 (0.22 mmol/1 g); IR (ν/cm^−1^): 3445 (SiO_2_), 1638, 1540, 1214–1029 (SiO_2_), 806 (SiO_2_), 467 (SiO_2_).

**CSP-6-OH** CHN analysis: C 5.45; H 0.55; N 0.77 (0.21 mmol/1 g); IR (ν/cm^−1^): 3448 (SiO_2_), 1645, 1543, 1347, 1217–1034 (SiO_2_), 805 (SiO_2_), 729, 465 (SiO_2_).

**CSP-7-OH** CHN analysis: C 6.13; H 1.15; N 1.10 (0.22 mmol/1 g); IR (ν/cm^−1^): 3452 (SiO_2_), 1643, 1545, 1346, 1235–1023 (SiO_2_), 805 (SiO_2_), 731, 460 (SiO_2_).

**CSP-8-OH** CHN analysis: C 6.73; H 1.17; N 0.41 (0.24 mmol/1 g); IR (ν/cm^−1^): 3469 (SiO_2_), 1638, 1545, 1348, 1230–1034 (SiO_2_), 807 (SiO_2_), 731, 463 (SiO_2_).

**CSP-9-OH** CHN analysis: C 5.36; H 0.63; N 0.96 (0.20 mmol/1 g); IR (ν/cm^−1^): 3451 (SiO_2_), 1636, 1544, 1350, 1228–1036 (SiO_2_), 807 (SiO_2_), 730, 468 (SiO_2_).

CSP-10-OH CHN analysis: C 6.41; H 1.07; N 1.06 (0.21 mmol/1 g); IR (ν/cm^−1^): 3439 (SiO_2_), 1630, 1542, 1347, 1223–1029 (SiO_2_), 806 (SiO_2_), 728, 464 (SiO_2_).

### 3.3. End-Capping and Packing Procedure

To a yellow suspension of prepared CSP-OHs in dry toluene (20 mL) in an inert atmosphere, hexamethyldisilazane (2 mL) was added and resulting mixture was refluxed for 20 h. The suspension was cooled, filtered using a G4 sinter and washed with toluene (30 mL) and methanol (2 × 30 mL). After drying of prepared CSP overnight at 60 °C, they were packed into stainless steel columns (150 × 4.6 mm I.D.) using the slurry packing technique (1.5 g of CSP was suspended in 25 mL of solvent hexane-2-propanol = 2:8).

### 3.4. Computational Methods

Minimum energy structures of **DNB-6**, **DNB-8** and **DNB-9** are taken from our previous publication [20]. Relaxed potential energy surface scan was performed in Gaussian 16 [43] with Minnesota global hybrid functional M06-2X [44] with 54% of the exact exchange and aug-cc-pVDZ functional. The ethenyl moiety of (*S*)-**DNB-6** from our previous work [20] was replaced by hydrogen. Ground state force field parameters of **CSP-6**, naproxen and acetic acid were evaluated using the *antechamber* module of the Amber 16 package and the generalized Amber force field (GAFF) [45]. After geometry optimization of the complex of (*S*)-**DNB-6** and naproxen (**A**) and (*S*)-**DNB-6**, naproxen and acetic acid (**B**), molecular dynamics simulations at 300 K in vacuum were run, with a time step of 1 fs and a time simulation of 10 ns, for both the (*S*)- and (*R*)-enantiomers of naproxen. Geometries were saved every 5 ps. The 20 lowest energy structures from the trajectories were optimized at the B3LYP/def-SVP level of theory. The 10 most stable complexes were then re-optimized using the same functional and larger basis set (def2-TZVPP), while final energies were evaluated on M06-2X/def2-TZVPP level with solvation effects incorporated via COSMO model [46] and dielectric constant that equals to 2.78. Geometry optimizations and single point energy calculations were run in Turbomole [47].

## 5. Conclusions

In conclusion, we prepared ten new Pirkle-type chiral stationary phases based on (*S*)-N-(1-aryl-propyl)-3,5-dinitrobenzamide selectors. We evaluated the prepared CSPs using thirty diverse racemates, including several nonsteroidal anti-inflammatory and 3-hydroxybenzodiazepine drugs. Our aim was to design CSPs with similar versatility to Whelk-O1 and three of our CSPs (**CSP-6**, **CSP-7** and **CSP-10**) indeed displayed chromatographic behavior comparable to this widely used CSP. The prepared CSPs differ in the aromatic unit directly linked to the chiral center, which enabled us to elucidate the influence of the size and substitution of aromatic moiety on the enantiorecognition process. In order to substantiate experimental results, we investigated the role of nonbonding interactions relevant for chiral recognition on CSPs using computational methods. The model system was enantioseparation of naproxen on **CSP-6** where we elucidated the influence of hydrogen bond network and aromatic interactions on enantiorecognition process. Furthermore, the stability of the complexes is in accordance with the experimentally determined elution order. Finally, we investigated the influence of acetic acid as an additive in the mobile phase in the abovementioned system. To the best of our knowledge, this study is the first one to investigate the possibility of positioning a minor additive within the chiral binding site. We showed that non-enantiospecific interactions with the carboxylic moiety of the analyte can change the interaction arrangements and influence the energetics of the complexes responsible for chiral recognition.

## Figures and Tables

**Table 1:** Comparison of the separation factors (α) of tested pharmaceutical drugs obtained on **CSP-6**, **CSP-7**, **CSP-10** and Whelk-O1 ^1^.

Compound	Whelk-O1	CSP-6	CSP-7	CSP-10
Ibuprofen	1.71	1.00	1.50	1.19
Ketoprofen	1.30	1.10	1.20	1.12
Naproxen	1.48	1.82	2.39	2.14
Flurbiprofen	1.68	1.14	1.53	1.27
Suprofen	1.27	1.11	1.16	1.11
Fenoprofen	1.60	1.26	1.55	1.24
Lorazepam	2.29	1.93	2.54	2.48
Oxazepam	1.97	1.39	1.59	1.59
Temazepam	1.38	1.31	1.39	1.45

^1^ For the analyses (*S*,*S*)-Whelk-O1 column (5 µm particle size, 250 × 4.6 mm I.D.) was used.

## Supplementary Materials

The Supplementary Materials are available online. Table S1: Enantioseparation results of racemates **1**–**7** and **9** on **CSP-1**–**CSP-10**, Table S2: Enantioseparation results of racemates **8** and **10**–**14** on **CSP-1**–**CSP-10**, Table S3: Enantioseparation results of racemates **15**–**21** on **CSP-1**–**CSP-10**, Table S4: Enantioseparation results of NSAIDs on **CSP-6**–**CSP-10**, Table S5: Enantioseparation results of 3-hydroxy-benzodiazepine drugs on **CSP-6**–**CSP-10**, Figure S1: Enantioseparation of naproxen on **CSP-6**, Figure S2: Higher energy structures of (*S*)-**A** and (*R*)-**A**, Figure S3: Higher energy structures of (*S*)-**B** and (*R*)-**B**.
