# Supplementary material for: New Brush-Type Chiral Stationary Phases for Enantioseparation of Pharmaceutical Drugs
Source: Molecules. 2019 Feb 25;24(4):823. doi: 10.3390/molecules24040823 (PMC6412842; doi:10.3390/molecules24040823)
Supplement: Supplementary file 1 [file molecules-24-00823-s001.pdf]

SUPPLEMENTARY MATERIALS

Novel brush-type chiral stationary phases for enantioseparation of pharmaceutical drugs

Anamarija Knežević [1,*], Jurica Novak [2] and Vladimir Vinković [1]

1 Division of Organic Chemistry and Biochemistry, Ruđer Bošković Institute, Bijenička cesta 54, Zagreb, Croatia

2 Division of Physical Chemistry, Ruđer Bošković Institute, Bijenička cesta 54, Zagreb, Croatia

Table S1. Enantioseparation results of racemates **1** – **7** and **9** on **CSP-1** – **CSP-10**; mobile phase: hexane-2-PrOH = 90 : 10; flow: 1 mL/min; UV detection at 254 nm. Column dimensions were 150 x 4.6 mm I.D., except for **CSP-1** which had the dimensions of 250 x 4.6 mm I.D.

**Compound**		**1**	**2**	**3**	**4**	**5**	**6**	**7**	**9**
**CSP-1**	k_2	1.28	4.67	2.84	2.90	1.72	2.83	3.25	9.03
	α	1.32	1.17	1.00	1.00	1.30	1.25	1.48	1.11
	R_s	2.16	1.76	0.00	0.00	2.45	2.18	3.20	1.61
**CSP-2**	k_2	0.29	0.97	0.69	0.75	0.52	0.58	0.90	1.37
	α	1.26	1.19	1.00	1.00	1.23	1.19	1.48	1.08
	R_s	0.89	0.96	0.00	0.00	0.87	0.97	3.32	0.94
**CSP-3**	k_2	0.15	1.23	0.70	0.88	0.36	2.64	1.30	1.42
	α	1.00	1.00	1.00	1.00	1.00	1.00	1.17	1.00
	R_s	0.00	0.00	0.00	0.00	0.00	0.00	1.39	0.00
**CSP-4**	k_2	0.12	0.83	0.36	0.40	0.36	0.21	0.37	0.72
	α	1.33	1.00	1.00	1.00	1.13	1.00	1.46	1.08
	R_s	0.47	0.00	0.00	0.00	0.47	0.00	1.36	0.62
**CSP-5**	k_2	0.09	0.27	0.20	0.25	0.30	0.17	0.30	0.37
	α	1.00	1.00	1.00	1.00	1.00	1.00	1.61	1.00
	R_s	0.00	0.00	0.00	0.00	0.00	0.00	1.30	0.00
**CSP-6**	k_2	0.70	2.72	1.68	1.82	1.26	1.36	1.51	3.11
	α	1.43	1.27	1.00	1.00	1.34	1.05	1.20	1.13
	R_s	2.82	2.55	0.00	0.00	2.95	0.49	2.23	2.18
**CSP-7**	k_2	0.72	2.57	1.35	1.71	1.44	0.94	1.38	2.76
	α	1.60	1.57	1.00	1.00	1.49	1.14	1.10	1.14
	R_s	4.13	4.43	0.00	0.00	4.28	1.05	0.90	2.36
**CSP-8**	k_2	0.37	1.01	0.68	0.91	0.51	0.74	1.45	1.50
	α	1.29	1.10	1.00	1.03	1.16	1.14	1.84	1.00
	R_s	1.43	0.46	0.00	0.39	0.69	0.82	4.98	0.00
**CSP-9**	k_2	0.35	1.13	0.89	0.98	0.71	0.73	0.89	1.87
	α	1.24	1.14	1.04	1.00	1.27	1.13	1.23	1.05
	R_s	1.18	1.06	0.49	0.00	1.86	0.90	1.62	0.69
**CSP-10**	k_2	0.69	2.24	1.51	1.84	1.74	1.17	1.10	2.74
	α	1.45	1.25	1.05	1.06	1.46	1.00	1.21	1.03
	R_s	2.88	1.09	0.59	0.64	3.15	0.00	1.44	0.47

Table S2. Enantioseparation results of racemates **8** and **10** – **14** on **CSP-1** – **CSP-10**; mobile phase: hexane-2-PrOH = 80 : 20; flow: 1 mL/min; UV detection at 254 nm. Column dimensions were 150 x 4.6 mm I.D., except for **CSP-1** which had the dimensions of 250 x 4.6 mm I.D.

**Compound**		**8**	**10**	**11**	**12**	**13**	**14**
**CSP-1**	k_2	7.03	2.77		22.37	2.27	11.85
	α	1.57	1.52	1.27	1.46	1.56	1.32
	R_s	4.20	3.76		3.94	3.76	3.28
**CSP-2**	k_2	1.90	1.18	2.30	8.00	1.12	2.45
	α	1.54	1.49	1.36	1.54	1.41	1.09
	R_s	4.83	2.88	3.85	4.38	3.54	1.00
**CSP-3**	k_2	3.00	0.54	1.68	2.04	0.85	9.12
	α	1.22	1.08	1.11	1.07	1.22	1.21
	R_s	1.47	0.47	0.87	0.59	1.51	1.52
**CSP-4**	k_2	0.84	0.61	1.02	2.61	0.50	0.72
	α	1.46	1.66	1.42	1.40	1.48	1.30
	R_s	2.84	2.46	3.08	3.47	2.36	1.85
**CSP-5**	k_2	0.33	0.21	0.41	0.82	0.17	0.37
	α	1.14	1.00	1.16	1.23	1.18	1.00
	R_s	0.59	0.00	0.76	1.61	0.49	0.00
**CSP-6**	k_2	4.21	2.61	4.51	11.79	2.16	14.10
	α	1.32	2.12	1.61	1.49	1.55	1.74
	R_s	3.56	6.68	7.38	4.91	6.57	3.44
**CSP-7**	k_2	4.08	3.60	5.56	16.94	2.40	11.68
	α	1.57	2.82	1.90	1.68	1.64	1.48
	R_s	6.16	9.05	9.80	5.56	7.93	4.93
**CSP-8**	k_2	2.33	0.88	2.26	6.80	1.70	3.78
	α	1.50	1.23	1.27	1.35	1.50	1.34
	R_s	4.91	1.50	3.21	3.30	5.22	4.13
**CSP-9**	k_2	2.78	1.64	2.98	8.02	1.41	6.94
	α	1.43	1.57	1.35	1.43	1.35	1.09
	R_s	5.38	6.00	5.34	5.36	4.25	1.25
**CSP-10**	k_2	4.72	2.96	4.36	9.92	1.69	19.14
	α	1.67	3.02	1.83	1.46	1.50	1.44
	R_s	4.96	7.12	7.01	3.77	5.22	2.93

Table S3. Enantioseparation results of racemates **15** – **21** on **CSP-1** – **CSP-10**; mobile phase: hexane-2-PrOH = 80 : 20; flow: 1 mL/min; UV detection at 254 nm. Column dimensions were 150 x 4.6 mm I.D., except for **CSP-1** which had the dimensions of 250 x 4.6 mm I.D.

**Compounds**		**15**	**16**	**17**	**18**	**19**	**20**	**21**
**CSP-1**	k_2	5.07	5.17	3.93	4.73	8.54	7.00	7.70
	α	1.07	1.06	1.10	1.06	1.15	1.04	1.07
	R_s	0.65	0.45	0.92	0.54	1.95	0.35	0.66
**CSP-2**	k_2	0.96	0.93	0.87	0.93	1.29	1.19	1.17
	α	1.08	1.10	1.29	1.15	1.00	1.14	1.19
	R_s	0.68	0.87	2.25	1.35	0.00	1.37	1.82
**CSP-3**	k_2	5.27	3.64	3.58	3.09	4.78	5.87	4.78
	α	1.14	1.19	1.17	1.19	1.06	1.06	1.17
	R_s	1.09	1.47	1.38	1.25	0.54	0.51	1.34
**CSP-4**	k_2	0.29	0.28	0.22	0.24	0.41	0.31	0.32
	α	1.00	1.00	1.00	1.00	1.16	1.00	1.00
	R_s	0.00	0.00	0.00	0.00	0.65	0.00	0.00
**CSP-5**	k_2	0.21	0.21	0.15	0.17	0.28	0.23	0.20
	α	1.00	1.00	1.00	1.00	1.17	1.00	1.00
	R_s	0.00	0.00	0.00	0.00	0.59	0.00	0.00
**CSP-6**	k_2	4.59	4.72	4.83	5.89	6.44	5.63	5.45
	α	1.31	1.43	1.65	1.52	1.03	1.27	1.41
	R_s	2.66	3.54	4.21	3.02	0.31	2.04	3.10
**CSP-7**	k_2	3.88	4.45	4.21	4.34	6.39	5.69	5.19
	α	1.31	1.48	1.81	1.51	1.10	1.37	1.45
	R_s	3.89	5.55	8.71	5.02	1.16	4.28	5.47
**CSP-8**	k_2	1.47	1.50	1.21	1.42	2.44	1.99	1.78
	α	1.00	1.09	1.12	1.07	1.23	1.13	1.09
	R_s	0.00	1.01	1.33	0.69	2.77	1.77	1.07
**CSP-9**	k_2	3.57	3.53	4.18	3.61	4.57	4.83	4.60
	α	1.45	1.51	2.08	1.64	1.18	1.45	1.67
	R_s	5.90	6.57	11.72	7.29	2.63	5.71	7.81
**CSP-10**	k_2	6.69	6.39	6.11	6.21	10.06	9.14	8.92
	α	1.19	1.28	1.50	1.33	1.06	1.32	1.36
	R_s	1.57	2.27	4.14	2.31	0.53	2.40	2.86

Table S4. Enantioseparation results of NSAIDs on **CSP-6** – **CSP-10**; flow: 1 mL/min; UV detection at 254 nm; column dimensions: 150 x 4.6 mm I.D.; mobile phase: top - hexane-2-PrOH-CH_3COOH = 90:10:0.1; bottom - hexane-2-PrOH = 80:20 + 1 g dm^{-3} NH_4OAc.

		Ibuprofen	Ketoprofen	Naproxen	Flurbiprofen	Suprofen	Fenoprofen
**CSP-6**	k_2	0.28	2.51	2.79	0.63	4.16	0.62
	α	1.00	1.00	1.34	1.06	1.00	1.08
	R_s	0.00	0.00	3.78	0.41	0.00	0.53
**CSP-7**	k_2	0.24	2.33	4.35	0.52	3.79	0.52
	α	1.14	1.05	1.58	1.14	1.00	1.21
	R_s	0.47	0.60	5.80	0.73	0.00	1.18
**CSP-8**	k_2	0.12	1.34	0.99	0.31	2.25	0.31
	α	1.00	1.00	1.00	1.00	1.00	1.00
	R_s	0.00	0.00	0.00	0.00	0.00	0.00
**CSP-9**	k_2	0.16	1.45	1.24	0.32	2.22	0.36
	α	1.00	1.00	1.09	1.00	1.00	1.00
	R_s	0.00	0.00	0.95	0.00	0.00	0.00
**CSP-10**	k_2	0.22	2.28	2.89	0.53	4.09	0.50
	α	1.00	1.00	1.45	1.00	1.00	1.10
	R_s	0.00	0.00	3.52	0.00	0.00	0.56
**CSP-6**	k_2	0.45	6.25	8.95	2.00	11.01	1.14
	α	1.00	1.10	1.82	1.14	1.11	1.26
	R_s	0.00	0.75	4.37	1.09	0.63	2.10
**CSP-7**	k_2	0.16	1.31	3.51	0.43	2.03	0.45
	α	1.50	1.20	2.39	1.53	1.16	1.55
	R_s	0.62	1.43	7.70	1.83	1.18	1.95
**CSP-8**	k_2	0.06	0.67	0.64	0.17	1.05	0.18
	α	1.00	1.00	1.08	1.00	1.00	1.00
	R_s	0.00	0.00	0.51	0.00	0.00	0.00
**CSP-9**	k_2	0.11	0.81	1.03	0.23	1.22	0.25
	α	1.00	1.00	1.21	1.00	1.00	1.00
	R_s	0.00	0.00	1.59	0.00	0.00	0.00
**CSP-10**	k_2	0.31	1.83	3.35	0.67	2.94	0.63
	α	1.19	1.12	2.14	1.27	1.11	1.24
	R_s	0.51	0.87	4.68	1.37	0.78	1.13

Table S5. Enantioseparation results of 3-hydroxy-benzodiazepine drugs on **CSP-6** – **CSP-10**; flow: 1 mL/min; UV detection at 254 nm; column dimensions: 150 x 4.6 mm I.D.; mobile phase: hexane-2-PrOH = 80:20 + 1 g dm^{-3} NH_4OAc.

		Lorazepam	Oxazepam	Temazepam
**CSP-6**	k_2	14.20	9.78	14.34
	α	1.93	1.39	1.31
	R_s	6.38	3.08	3.13
**CSP-7**	k_2	5.44	3.13	6.31
	α	2.54	1.59	1.39
	R_s	8.11	4.28	3.87
**CSP-8**	k_2	1.94	1.45	2.67
	α	1.47	1.20	1.12
	R_s	3.63	1.34	1.24
**CSP-9**	k_2	2.46	1.82	3.83
	α	1.53	1.17	1.14
	R_s	4.84	1.66	1.53
**CSP-10**	k_2	6.59	4.27	8.61
	α	2.48	1.59	1.45
	R_s	6.00	1.89	3.02

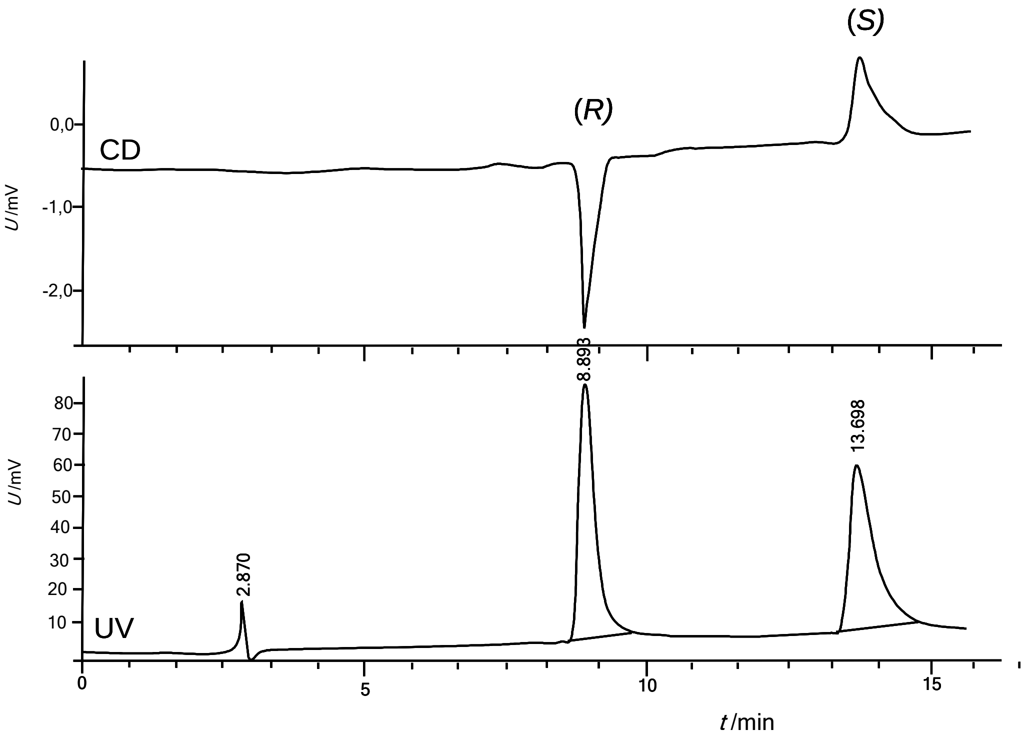

Figure S1. Enantioseparation of naproxen on **CSP-6**, column dimensions 250 x 4.6 mm I.D., mobile phase hexane-2-propanol = 80:20 + 1 g dm^{-3} NH_4OAc, flow 1mL/min, UV detection at 271 nm.

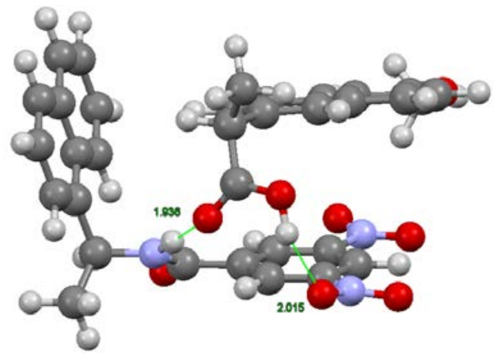

S-**A**-2 0.16

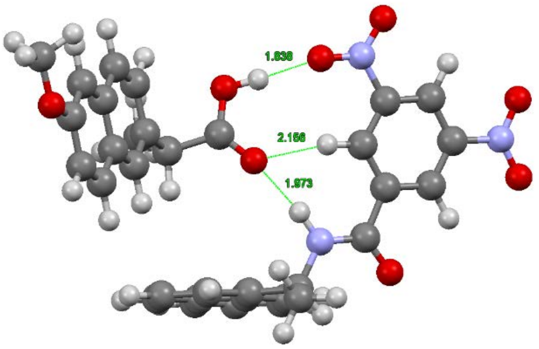

R-**A**-2 2.61

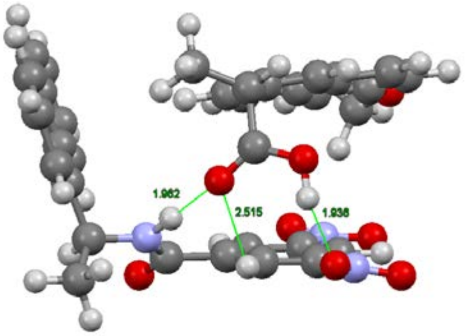

S-**A**-3 0.37

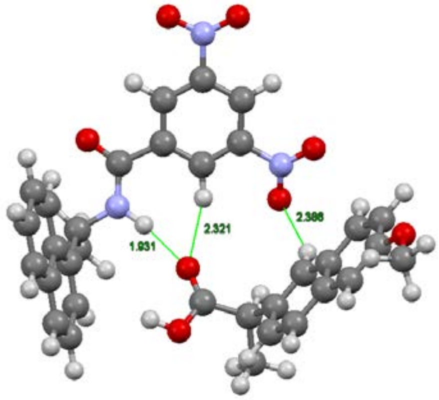

R-**A**-3 2.63

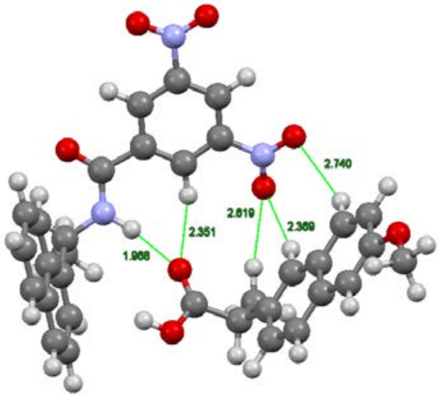

S-**A**-4 2.45

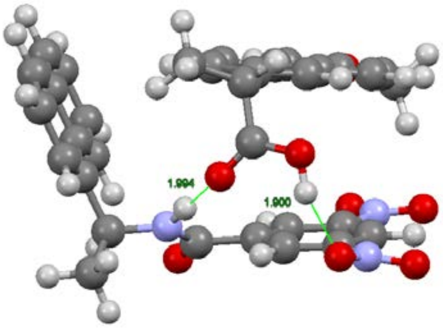

R-**A**-4 2.77

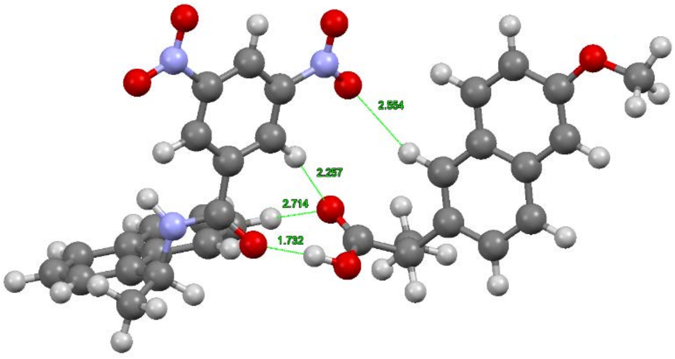

S-**A**-5 2.62

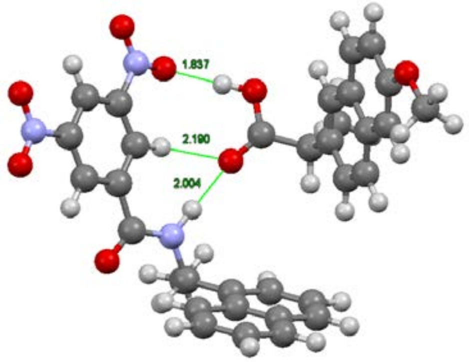

R-**A**-5 2.86

Figure S2. Higher energy structures of *S*-**A** (left) and *R*-**A** (right). Relative energies (in kcal mol^{-1}) to *S*-**A** are shown. B3LYP/def2-TZVPP// M06-2X/def2-TZVPP (COSMO, ε=2.78).

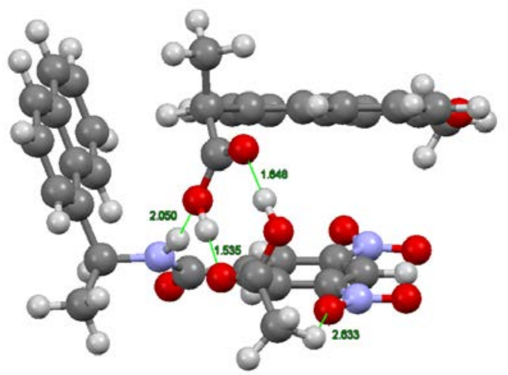

S-**B**-2 1.08

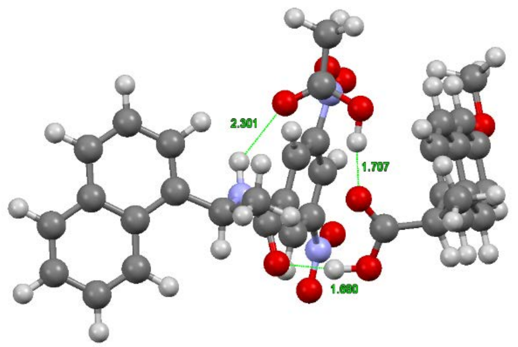

R-**B**-2 4.96

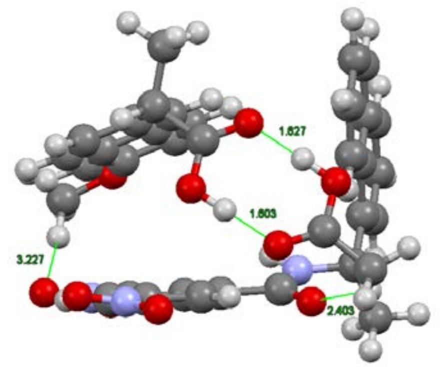

S-**B**-3 3.83

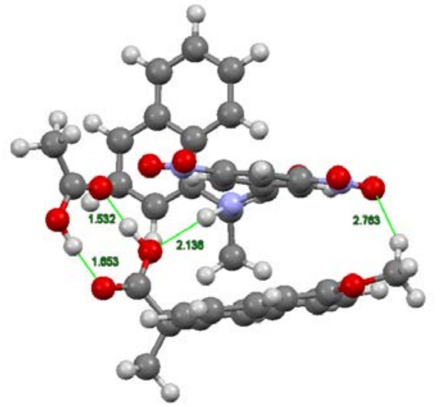

R-**B**-3 4.97

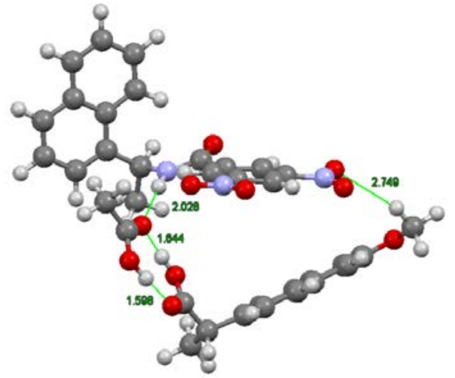

S-**B**-4 3.78

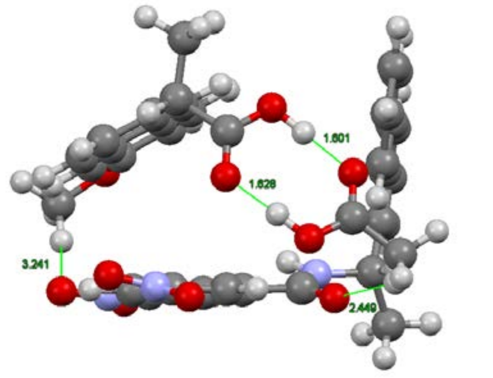

R-**B**-4 5.97

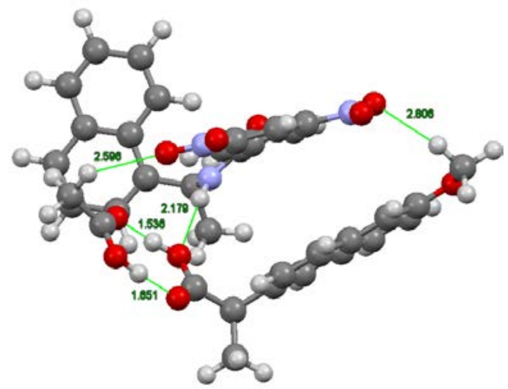

S-**B**-5 4.79

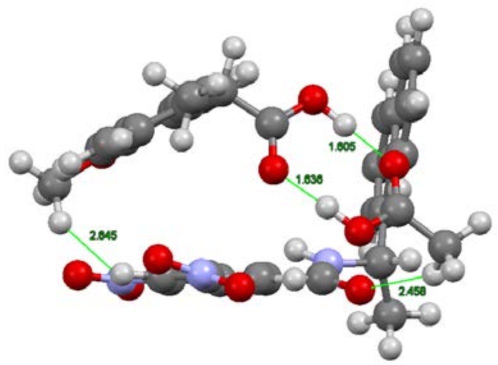

R-**B**-5 6.79

Figure S3. Higher energy structures of *S*-**B** (left) and *R*-**B** (right). Relative energies (in kcal mol^{-1}) to *S*-**B** are shown. B3LYP/def2-TZVPP// M06-2X/def2-TZVPP (COSMO, ε=2.78).
